# Carbonized polymer dots enhanced stability and flexibility of quasi-2D perovskite photodetector

**DOI:** 10.1038/s41377-022-01000-6

**Published:** 2022-10-18

**Authors:** Mingrui Tan, Mingbian Li, Wanting Pan, Xiaopeng Feng, Yuhong He, Junjun Liu, Fengxia Dong, Haotong Wei, Bai Yang

**Affiliations:** 1 State Key Laboratory of Supramolecular Structure and Materials, College of Chemistry, Jilin University, 130012 Changchun, China; 2 Optical Functional Theranostics Joint Laboratory of Medicine and Chemistry, The First Hospital of Jilin University, 130012 Changchun, China

**Keywords:** Optoelectronic devices and components, Nanoparticles

## Abstract

Quasi-2D perovskites have been demonstrated to be competitive materials in the photodetection fields due to the enhanced moisture stability by large organic cations. However, as the increasing demands of modern technology, it is still challenging to combine the flexibility with the capability of weak light detection in a low-cost way. Here, amides, carboxylic acids, and anhydrides groups-rich carbonized polymer dots (CPDs) were employed to fill in the perovskite grain boundaries, which can passivate the point defects of perovskite by coordinating with the unbonded Pb atoms, and reduce the leakage current. Weak light detection capability was demonstrated by directly resolving light with an intensity of 10.1 pW cm^−2^. More importantly, the stretchable polymer chains on CPDs strongly interact with perovskite ions through multiple supramolecular interactions, and extend the stretchable properties to the perovskite/CPDs composites, which can maintain the integral structure stability during the deformation of perovskite crystals and restricted any crack by releasing the film strain. Our fabricated devices show extraordinary flexible stability in the bending-dependent response tests. The viscoelasticity of CPDs improves the bending stability of the flexible quasi-2D perovskite photodetectors, and device performance shows no degradation after bending 10000 times, comparable or even outperforming the dominating flexible photodetectors.

## Abstract (web summary)

Carbonized polymer dots effectively improved the stability and flexibility of perovskite photodetector by achieving no loss response after bending 10000 times and resolving a weak light of 10.1 pW cm^−2^

## Graphical abstract

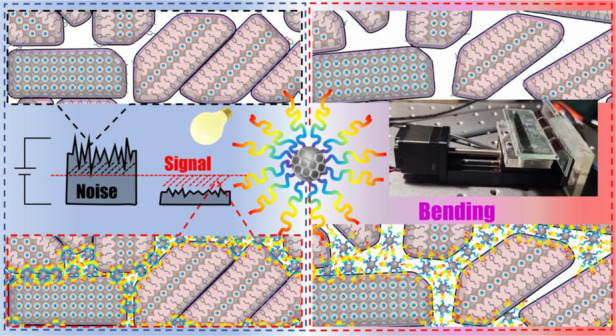

## Introduction

High-performance photodetectors with great detection capability have been widely deployed in our daily life, such as driverless technology, intraoperative navigated surgery, face recognition, anti-counterfeiting, and so on. However, we still face challenges as new demands are required for additional functions like excellent flexibility with no sensitivity/stability loss. Therefore, novel photosensitive materials or multi-functional composites are needed to meet the fast technology development. Perovskites as excellent optoelectronic semiconductors have attracted great interest in interdisciplinary fields such as energy conversion^1–3^, light emitting diodes^4,5^, displays^6,7^, ionizing radiation detection, and UV-visible light detection^8–12^, and so on. The attractive properties such as low-cost solution processes^13–15^, low nucleation formation energy^16,17^, great defects tolerance feature^18–21^, and the extraordinary charge carrier kinetics^22–26^ enable perovskites as ideal optoelectronic materials for photodetection applications. However, due to the low forming energy of the three-dimensional organic-inorganic hybrid perovskites such as methylammonium lead iodide (MAPbI_3_), formamidine lead iodide (FAPbI_3_), and methylamine tin iodide (MASnI_3_), their moisture instability or thermal instability limit their further commercialization^27–29^. To solve these issues, low dimensional perovskites are a good choice by separating the inorganic framework with large organic cations. 2D/quasi-2D perovskites have been demonstrated to be good candidates with improved device stability^11,30,31^. Furthermore, to expand the function of perovskite materials, composite materials were designed by combining the advantages of perovskite with other materials like MXene, metal-organic framework, and so on^32–37^. The optoelectronic device’s performance and stability can be largely improved. However, the rigid nature of these materials limits the flexibility of the perovskite devices.

Carbonized polymer dots (CPDs) are composed of carbonized crystalline carbon as a nucleus core in the center and capped by amorphous polymer chains as shells. The size of the carbon nucleus can be easily controlled by modulating the carbonized temperature and time, and the polymer chains possess abundant functional groups, which bring tunable surface chemical properties. Therefore, the unique core-shell structure of CPDs ensures excellent viscoelasticity and tunable optoelectronic properties of CPDs. In addition, the raw materials of CPDs are abundant in nature, and synthesis processes are also facile and low cost. Based on these considerations, we proposed that perovskite/CPDs composites may exhibit excellent photodetection performance and flexibility.

In this manuscript, we developed three different 4-fluorophenethylamine based methylammonium lead iodide (FPEA_2_MA_3_Pb_4_I_13_, FMPI) perovskite/CPDs composites for flexible photodetectors application. The CPDs distribute at the grain boundaries of FMPI material, passivate the surface defects of the FMPI grain, and cross-link the grains with coordinative interactions between electrical negative functional groups and the unbonded Pb atoms. The passivation effect of CPDs reduces the device leakage current and improves the capability of weak light detection. Functional groups, including amides, carboxylic acids, and anhydrides, were gradiently distributed on the polymer chains by increasing the carbonation degree of CPDs. The viscoelasticity of the polymer chains facilitates maintaining the perovskite grains’ integrity and film uniformity during deformation and improves the bending stability of the flexible FMPI/CPDs composites. The flexible FMPI/CPDs photodetector shows no photocurrent signal loss after 10000 times bending, and simultaneously resolves weak light intensity of 10.1 pW cm^−2^, comparable or even outperforming the state-of-the-art photodetectors.

## Results

### Coordinative groups design on polymer chains of CPDs

**Fig. 1:**
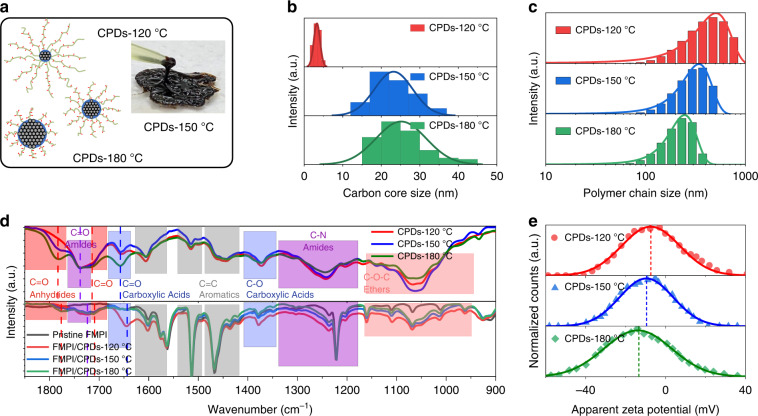
Morphology and functional group characteristics of CPDs. **a** The diagram for morphologic properties of various CPDs fabricated under different carbonization temperatures. Insert: photograph of fabricated viscoelastic CPDs. The distributions of the carbon cores (**b**) and polymer chains (**c**) analyzed from TEM and DLS measurements, respectively. **d** The FTIR spectrum of different CPDs (top) and FMPI/CPDs (bottom). **e** The zeta potential pattern of three different CPDs

The CPDs were fabricated according to our previous method^38^. To obtain functional CPDs with various coordinative behavior and carbon nuclear sizes, the carbonization temperatures were set as 120 °C, 150 °C, and 180 °C, respectively as illustrated in Fig. 1a. Insert is the corresponding photo of synthesized CPDs, highlighting the viscoelastic property brought by their surface polymer chains. We employed transmission electron microscope (TEM) measurements to quantify the carbon core size of the three CPDs (Fig. S1), and the results are summarized in Fig. 1b. The average core sizes of the CPDs are 6 nm, 23 nm, and 25 nm, respectively, as temperature increases, indicating enhanced carbonization degree. We also used dynamic light scattering (DLS) measurements to characterize the polymer chain lengths of the CPDs, as shown in Fig. 1c. It should be noted that the DLS results are the hydration size of CPDs, which is normally larger than the actual size of the CPDs. CPDs-120 °C are around 500 nm in diameter, larger than that CPDs-150 °C and CPDs-180 °C of 350 nm and 240 nm, respectively. To further confirm the polymer chains sizes, Deionized water as a poor solvent was gradually added into the CPDs solutions. The polymer chains gradually shrink to the very surface of the carbon core in this case, as shown in Fig. S2, consistent with the TEM results.

To further specify the active functional groups on polymer chains of CPDs, we carried out the Fourier Transform Infrared Spectroscopy (FTIR) tests in Fig. 1d, and the characteristic peaks of amides, carboxylic acids, and anhydrides appear as functional groups on CPDs. By normalizing the FTIR spectra with the total carbon vibration, we discovered that anhydrides amount obviously increased at elevated temperatures as a result of enhanced carbonization degree. Anhydrides often result from the condensation reaction of carboxylic acids groups. However, the zeta potential analysis in Fig. 1e shows that the potential of CPDs-180 °C is −13.9 mV, higher than that of −7.4 and −9.6 mV of CPDs-120 °C and CPDs-150 °C, respectively. Since the number of carboxylic acids on CPDs-180 °C should be less than the other two, we attributed the higher potential of CPDs-180 °C to the shorter polymer chains and higher charges density, which is consistent with the DLS results. The functional groups on CPDs can bring coordination with the unbonded Pb defects at the perovskite grain boundaries, which is also observed in the FTIR spectra in Fig. 1d. Since the stretching vibration peaks intensity of amides and carboxylic acids groups are largely reduced, and the peaks of anhydrides groups almost disappear, showing the strong interactions between perovskite and CPDs.

### Interactions between CPDs and FMPI perovskites

**Fig. 2:**
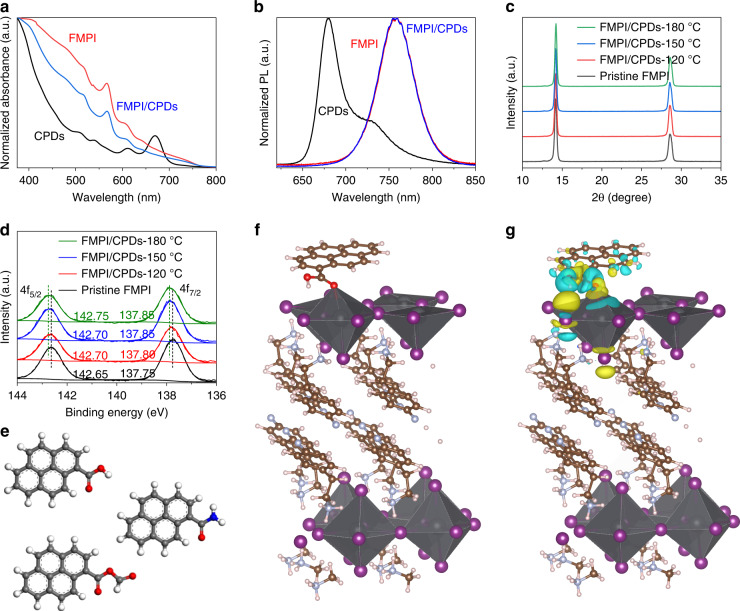
Interactions between FMPI and CPDs. **a** The absorbance and (**b**) photoluminescence spectrum of CPDs-120 °C, pristine FMPI, and FMPI/CPDs-120 °C. **c** XRD and (**d**) XPS pattern of pristine FMPI and FMPI/CPDs thin films. **e** Built brief structure of CPDs with carboxylic acids, amides, and anhydrides groups, respectively. **f** The optimized structure and (**g**) differential charge analyses of CPDs with carboxylic acid group coordinate with unbonded Pb atoms

To study the interactions between CPDs and FMPI perovskites, Perovskite/CPDs composites were fabricated by adding the CPDs into the perovskite solution and span-coated thin films. The absorbance spectra of the composite films are displayed in Fig. 2a, which are similar to that of pristine FMPI perovskite film due to the high absorption coefficients of perovskite. The absorption edge of the CPDs is located at 680 nm, indicating a larger bandgap than that of pristine FMPI. The photoluminescence (PL) spectra in Fig. 2b shows a narrow PL peak of CPDs, and this illustrates a clear band edge structure with fewer trap states. The absence of CPDs PL emission from the FMPI/CPDs thin film shows possible charges/energy transfer from CPDs to FMPI. The X-ray Diffraction (XRD) study in Fig. 2c demonstrates that no obvious phase separation is observed after including CPDs in FMPI films, and crystalline intensity and behavior remain the same with pristine FMPI film. These results also reveal that the CPDs mainly distribute at the grain boundaries of the FMPI perovskite, which should maintain its origin optoelectronic properties.

To further clarify the mechanism of the interactions between the CPDs and the FMPI perovskite, we explored the specific interaction between the CPDs and the FMPI perovskite via x-ray photoelectron spectroscopy (XPS) measurements in Fig. 2d. Considering the electrical negative functional groups densities follow the order of *ρ*(CPDs-180 °C) > *ρ*(CPDs-150 °C) > *ρ*(CPDs-120 °C), the stronger coordination interactions by passivating Pb atoms will lead to an obvious increase in the binding energy of Pb 4*f* electrons and a larger shift in XPS spectra.

Meanwhile, we adopted density function theory (DFT) calculation to further analyze the interactions between the three CPDs and the FMPI perovskites. Considering the computing complexity and available resources, we simplified the CPDs model to simulate their interaction with the FMPI perovskite by establishing a pyrene with a specific functional group, as shown in Fig. 2e. We built the FMPI structure with Pb-I exposed at the surface to represent the Pb defects at the grain boundaries. Figure 2f shows the optimized structure of carboxylic acid-ended CPDs adsorbs on the Pb-I surface. The Oxygen atom of the carbonyl locates near the Pb atom, showing a coordination effect. Further charges difference was calculated to analyze the charge transfer properties. As shown in Fig. 2g, the yellow area distributes around the O atom, indicating an accumulation of electrons, which are transferred from the Pb atoms as the blue area illustrated. The structures comprised of FMPI/CPDs based on amides and anhydrides are also optimized and analyzed, respectively, as seen in Figure S4 and S5, showing similar results.

The binding energy of the three types CPDs coordinating with the Pb surface was calculated by using the following equation:

1 $$E_{{\rm{bin}}} = E_{{\rm{total}}} - E_{{\rm{FMPI}}} - E_{{\rm{CPDs}}}$$

where *E*_bin_ represents the binding energy between perovskite and CPDs, the *E*_total_ represents the energy of the CPDs coordinated FMPI system, the *E*_FMPI_ and *E*_CPDs_ represent the calculated energy of pristine FMPI and CPDs structure, respectively. The *E*_bin_ of perovskite with amides, carboxylic acids, and anhydrides ended CPDs were −0.50, −0.35, and −0.25 eV, respectively. The larger value of *E*_bin_ illustrates, the more stable the structure is. Therefore, the CPDs-180 °C interact with the Pb atom the most strongly, further confirming the binding energy shift of the 4*f* electrons on Pb atoms.

### Enhanced flexibility and stability of FMPI/CPDs composites

**Fig. 3:**
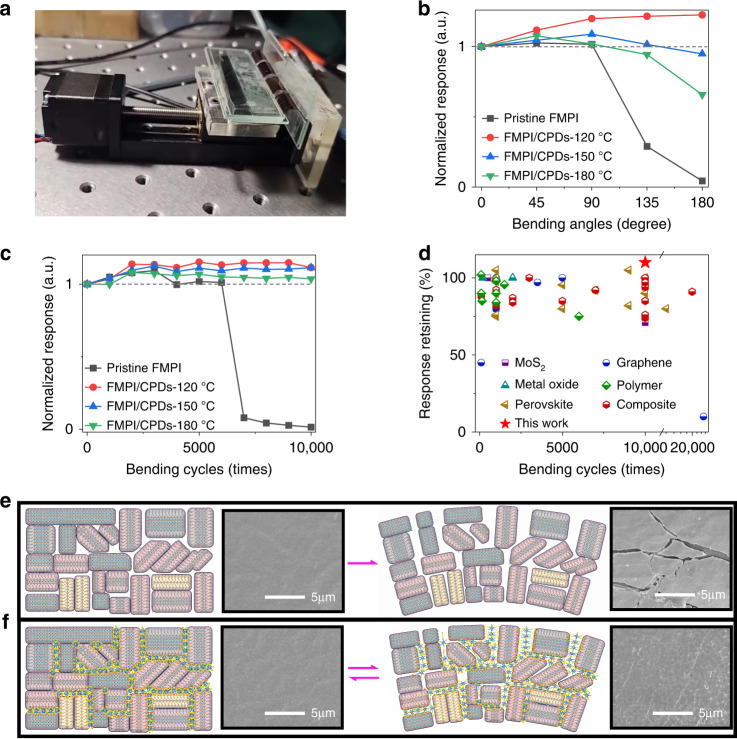
Flexibility and stability of FMPI/CPDs photodetectors. **a** Photo of the homemade motor bending system. The flexible stability of various bending angles (**b**) and bending times (**c**). **d** Plots of bending stability for flexible optoelectronic devices reported. **e** Schematic diagram of pristine FMPI thin film before and after bending process, with SEM images alongside. **f** Schematic diagram of pristine FMPI/CPDs thin film before and after bending process, with SEM images alongside

Considering the unique core-shell structure of the CPDs, we proposed that the crystalline carbon core favors the charges transfer and transport, and the viscoelasticity of polymer chains facilize the flexibility of the composite film once functional groups on the CPDs surface anchoring the perovskite boundaries. We further fabricated the flexible photodetector devices based on the FMPI/CPDs films and evaluated the device performance at various bending conditions. To bend the flexible devices, a cycle motor was employed, and different bending angles can be obtained by driving the motor to move different distances in Fig. 3a. All the devices were bent to 45°, 90°, 135°, and 180° angles 1000 times, and the on/off responsivity of the photodetector devices at various bending angles was measured as shown in Figs. 3b and S7. The modulated 532 nm light is employed with an intensity of 1.1 mW cm^−2^, and no bias was applied to the flexible devices. The pristine FMPI devices exhibit fast signal degradation of the response signal that with increasing once the bending angle is up to 135°, and the response signal falls to 30% of the initial response amplitude. Further increasing the folding degree to 180° will damage the device with a response signal lower than 5%. FMPI/CPDs composite devices show obviously enhanced flexibility and stability, although there is a little bit of degradation at 135° and around 70% signal remaining for the CPDs-180 °C sample. To compare the flexibility of our device with the existing ones, we bent our FMPI/CPDs photodetectors at a 90° angle 10000 times. There is no signal loss during the bending processes, as shown in Fig. 3c, and the control device without CPDs seriously degraded after 6000 times of bending. It should be noted that there is no encapsulation on our device during the bending tests. Figure 3d shows the summarized performance of reported flexible devices made of different materials, and we can conclude that the flexibility of our FMPI/CPDs is comparable or even better than the dominating flexible devices, as detailed in Table S1.

To explore the reason for the performance degradation of our flexible devices, scanning electron microscope (SEM) characterizations are performed, and corresponding degradation schemes are also described in Fig. 3e, f. The SEM images in Fig. 3e show the pristine FMPI perovskite device before and after bending. It can be clearly seen that there are lots of cracks on the film after 5000 times bending at a 90° angle. Grain boundaries are the locations where cracks will be generated after bending tests. In contrast, no cracks are observed for the FMPI/CPDs composite device under the same condition as shown in Fig. 3f, which benefits from the viscoelasticity properties of polymer chains on the CPDs surface.

### Photodetector performance of FMPI/CPDs composites

**Fig. 4:**
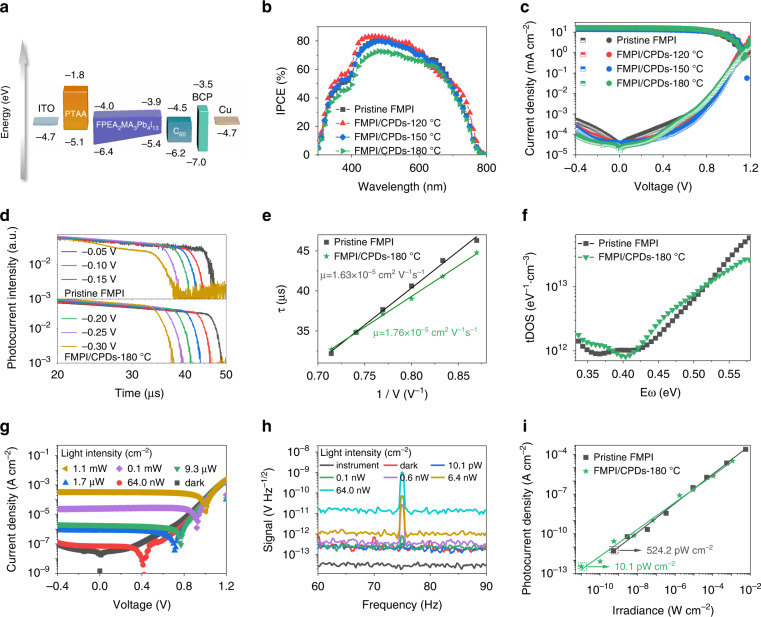
Performance and characteristics of FMPI/CPDs photodetectors. **a** Band structure of the pristine FMPI photodetector device. **b** IPCE response and (**c**) *I*–*V* curves of pristine FMPI and FMPI/CPDs photodetectors. **d** ToF transients and (**e**) the calculated mobility results of pristine FMPI and FMPI/CPDs-180 °C devices. **f** The trap density of state for pristine FMPI and FMPI/CPDs-180 °C photodetectors. The light-dependent response of FMPI/CPDs-180 °C photodetectors (**g**) and (**h**) NEP measurements. **i** The LDR results of pristine FMPI and FMPI/CPDs-180 °C photodetectors

Excellent flexibility does not guarantee excellent photodetector performance since device traps may generate during bending processes. The photodetector devices based on pristine FMPI and FMPI/CPDs thin films were fabricated with a device structure of ITO/PTAA/FMPI or (FMPI/CPDs)/C_60_/BCP/Cu as shown in Fig. 4a. Quasi-2D perovskite with a low n value (*n* < 4) is mainly distributed at the bottom of the perovskite thin film^39,40^, which facilities charges transportation in a p-i-n device structure.

The monochromatic incident photon-to-electron conversion efficiency (IPCE) of all the fabricated devices was carried out as shown in Fig. 4b, and similar IPCE spectra were obtained between the FMPI/CPDs devices and control device, showing comparable sensitivity of the FMPI/CPDs devices. This indicates that the introduction of CPDs does not impair the charges transport, consistent with the *I–V* measurements. The responsivity (*R*_es_) was calculated as follows:

2 $$R_{{\rm{es}}} = \frac{{{\rm{EQE}}{{{\mathrm{q}}}}}}{{h\nu }}$$

where *q* represents the electric quantity of an elementary charge, *hν* represents the energy of a single-photon incident on the device at the determined wavelength. The *R*_es_ is 0.42 A/W for pristine FMPI and 0.39 A/W for FMPI/CPDs-180 °C photodetector devices. The *I–V* measurements under dark and light conditions are directly carried out in Fig. 4c, and the FMPI/CPDs devices show similar photocurrents to the control device since all charges can be extracted under applied bias. However, the dark current varies as the different kinds of CPDs in the optoelectronic layer, and CPDs-120 °C present the largest leakage current, and CPDs-180 °C have the lowest dark current. The lower dark current would improve the signal/noise ratio to better resolve the weak light with a greater photodetection limit.

We further investigated the charge carrier kinetics via time of flight (ToF) measurements. The ToF transients of pristine FMPI and FMPI/CPDs-180 °C versus various bias voltages are listed in Fig. 4d. Obviously, the transient time shortens with increasing the extraction voltage for both devices. We statistics the changing tendency of transient time with the reciprocal voltage as listed in Fig. 4e. The mobility of both devices was quantified by fitting the lines and calculated through the equation:

3 $$\mu = \frac{v}{E} = \frac{{d^2}}{{\tau _{{\rm{tr}}} \times V}}$$

where *d* represents the film thickness of the light harvester layer, *v* represents the transfer velocity, *E* represents the applied electric field, *V* represents the applied voltage, and *τ*_tr_ represents the transient time. The calculated mobility value of pristine FMPI and FMPI/CPDs-180 °C is 1.63 cm^2^ V^−1^ s^−1^ and 1.76 × 10^−5^ cm^2^ V^−1^ s^−1^, respectively. The trap density of the state was also measured, as shown in Fig. 4f. The trap density of states was calculated via^41^

4 $$\normalsize \it \normalsize \normalsize \it \it N_t\left( {E_\omega } \right) = - \frac{{V_bdC\omega }}{{qWd\omega {{{\mathrm{k}}}}_BT}}$$

where *N*_t_ represents trap density of states, *E*_ω_ represents demarcation energy, *V*_b_ represents the build-in electrical potential, *q* represents the elementary charge, *W* represents the depletion region width, *ω* is the measuring angular frequency, *C* is the capacitance, *k*_B_ is the Boltzmann constant and *T* represents the thermal temperature. With 180 °C CPDs coordinated with the unbonded Pb atoms, the trap states with trap depth of 0.52–0.58 eV has been passivated. The lowered deep depth trap facilitates the charge carrier drifting process, which leads to higher mobility.

As the FMPI/CPDs-180 °C device holds the lowest dark current value, it would benefit from the capability to detect weak light. The photocurrent response under various light intensities was quantified via source meter at a bias voltage of −0.3 V. As illustrated in Fig. 4g, the photocurrent amplitude degrades with weakening the luminescence intensity. When reached the examining limit of the source meter, we further clarify that the revolution of photocurrent varies with weak illuminance by noise equivalent power (NEP) measurement via an amplifier and dynamic signal analyzer. As seen in Fig. 4h, the measurement equipment exists a system noise with the magnificence of 10^−14^ V Hz^−1/2^. The lowest detectable light intensity of the pristine FMPI photodetector device is 542.4 pW cm^−2^, while for the FMPI/CPDs-180 °C device, the detection limit was improved to 10.1 pW cm^−2^, as shown in Fig. 4i. The theoretical NEP value was calculated via

5 $${\rm{NEP}} = \frac{{i_n}}{{R_{{\rm{es}}}}}$$

while *i*_n_ and *R*_es_ represents the noise current amplitude and the responsivity, respectively. The calculated value was 5.7 pW Hz^−1/2^ for FMPI/CPDs-180 °C device, comparable to the directly measured value. The specific detectivity (D*) was also quantified according to the equation:

6 $$D^ \ast = \frac{{\sqrt {AB} }}{{{\rm{NEP}}}}$$

where *A* represents the effective area and *B* represents the bandwidth of the photodetector device. The D* value of pristine FMPI device is 4.82 × 10^9^ at 532 nm, lower than that of 6.21 × 10^10^ Jones for FMPI/CPDs-180 °C device. These results further confirm that the introduction of the CPDs improves the weak light detection limit of flexible perovskite film.

## Discussion

We developed three different FMPI/CPDs composites for thin-film photodetectors to modulate the flexibility and stability of composite films by combining the advantages of CPDs’ viscoelasticity. The functional groups on CPDs can coordinate with unbonded Pb atoms and passivate the defects of perovskite. The viscoelasticity of polymer chains on CPDs keeps the morphology stability of the perovskite during the deformation process and holds the optoelectronic performance with no reduction in photo-response after 10,000 times bending at 90°. Simultaneously, the coordinating CPDs suppress the device leakage current, enhance the detectivity of FMPI photodetectors, and directly resolve weak light with an intensity of 10.1 pW cm^−2^. The strategy provides an available path to boost the photodetection performance of flexible devices.

## Materials and methods

### Materials

The 2,9-dimethyl-4,7-diphenyl-1,10-Phenanthroline (BCP, >99% HPLC), lead iodide (PbI_2_, ≥99.99%), Poly[bis(4-phenyl)(2,4,6-trimethylphenyl)amine] (PTAA, Mw: 1000-10000 by GPC) and Poly(3,4-ethylenedioxythiophene) polystyrene sulfonate (PEDOT:PSS, AI 4083) were purchased from Xi’an Polymer Light Technology Corp. Methylamine hydroiodide (MAI, 99%) was purchased from Great Cell. 4-fluorophenethylammonium (FPEA, 99%), Hydroiodic acid (HI, 55–58%), and N, N-Dimethylformamide anhydrous (DMF, 99.9%) were purchased from Energy Chemical. All materials were used as received without any further purification.

### Synthesis of FPEAI organic ammonium cations

The FPEA was mixed into ethanol, and then the HI solution was dropwise into the solution under ice bath conditions. The molar ratio of FPEA and HI was 1:1. The solution was stirred for 2 h to make the process fully react, followed by rotary evaporation to obtain crude products. The powder was washed with ether three times and dried in a vacuum oven to remove the organic solvent. The FPEAI organic ammonium salt was stored in a glove box for further use.

### Synthesis of carbonized polymer dots

The CPDs were synthesized via solvothermal procedure by smashing Taxus leaves into powder and dissolved in acetone solution with a concentration of 50 mg/mL. The solution was transferred into a Teflon-lined autoclave and heated in the oven for 5 h at 120 °C, 150 °C, and 180 °C, separately. The mixture was centrifuged at 8000 rpm for 10 min, and the supernatant solution was collected, followed by removing the large particles through a 0.22 μm polyethersulfone membrane. The crude product was treated with dry silica column chromatography via a mixture of ethyl acetate and petroleum ether with a dynamic ratio to ensure the product would be effectively collected. The final CPDs product was obtained by vacuum distillation and stored in a glass desiccator.

### Fabrication of perovskite precursor solution

The pristine FMPI and FMPI with various CPDs dopped thin film was fabricated by one-step method via solution route. For the pristine FMPI precursor solution, the FPEAI, MAI, and PbI_2_ were dissolved in DMF solvent with a molar ratio of 2:3:4. The concentration of Pb cations was 1 mmol/mL. The solution was stirred for 4 h under ambient temperature to ensure the salts had been fully dissolved. The solution was filtrated via a 0.22 μm polyethersulfone membrane. The various CPDs powder was straight dissolved in FMPI precursor solution with a concentration of 0.1 mg/mL (100 mg/mL for FTIR measurement and 5 mg/mL for XPS measurement). The FMPI and FMPI/CPDs precursor was stored in the glove box for further use.

### Fabrication of perovskite thin films

The FMPI and FMPI/CPDs thin films were fabricated via the one-step method. The treated substrates were transferred into the glove box under nitrogen conditions. Then 50 μL FMPI or FMPI/CPDs precursor solution was added to the substrate and spin-coated with an rpm of 4000 for 30 s after which the color changed from yellow to light brown. Then the substrate was transferred onto a hot plate and annealed with a temperature of 100 °C for 10 min and the thin film become dark brown.

### Fabrication of rigid and flexible photodetector devices

The rigid and flexible ITO substrate was washed under an ultrasound machine in deionized water, ethanol, acetone and isopropanol and dried in the oven. The rigid and flexible substrate was treated with plasma for 5 min. The PTAA (5 mg/mL in toluene) was spin-coated on the rigid ITO with 4000 rpm for 30 s and then annealed at 120°C for 5 min in a glove box. The PEDOT: PSS was spin-coated with 4000 rpm for 30 s and annealed at a hot plate at 120 °C for 30 min in an ultra-clean laminar airflow bench under ambient conditions. The substrates were transferred into a glove box and spin-coast FMPI precursor solution to form perovskite thin films. The electron transform material of C_60_, BCP, and copper electrodes was evaporated with a thickness of 20 nm, 8 nm, and 80 nm, respectively.

### Characterization

The pristine FMPI and FMPI with various CPDs powder was obtained by scratching down from the thin film samples, and the as-fabricated CPDs powder was used directly. All the powders were smashed with KBr salt with a weight ratio of 10% and tableted to form thin semi-transmittance plates separately. The FTIR spectrum was measured via an infrared spectrometer (Brucker IFS66V). The carbon core morphology information of the various CPDs was obtained via an analytical electron microscope (JEOL, JEM-2001F). The polymer chain size of the various CPDs was quantified by DLS measurement via a light scatting spectrometer (ALV, CGS-3). The UPS spectrum was carried out with an X-ray photoelectron spectrometer (VG ESCALab, Mark II). The absorbance spectrum was obtained with a UV–visible spectrophotometer (Shimadzu, 3600). The photoluminescence spectrum was brought out with a homemade PL measure system, containing a photon counter ranging from visible to infrared wavelength (Zolix). The XRD pattern was measured using an X-ray diffractometer (Rigaku, SmartLab) with Cu Kα radiation. The IPCE measurement was accomplished with an IPCE system (built up by Zolix). The *I–V* property was characterized under various light sources via a source meter (Keithley, 2400). The NEP measurement was achieved by using an amplifier (Stanford Research Systems, SR570) and a dynamic signal analyzer (Keysight, E35670A) to collect the response current signal. The light was modulated and co-frequency by a function signal generator (RIGOL, DG1000). The tDOS measurements were carried out with an LCR meter (Keysight E4980A) at illumination conditions.

### DFT calculation

The first-principles calculations were employed via the Vienna Ab initio Simulation Package. The electron-ion interaction was described using projected augmented-wave pseudopotentials with Perdew-Burke-Ernzerhof generalized gradient approximation as the exchange-correlation function. The atomic positions were fully optimized until the force on each atom was smaller than 0.01 eV, and the convergence threshold for the self-consistent field was 10^−4^ eV. The FMPI was built using a two-layer [PbI_6_]^4−^ framework with a PbI layer exposed at the surface. The carboxylic acids, amides, and anhydrides-based CPDs binding on the PbI layer to simulate the various functional groups of CPDs coordinated with the unbonded Pb atoms. The binding energy was calculated by achieving the self-consistent calculation of isolate FMPI, CPDs, and CPDs binding FMPI structure separately with the same crystal parameters.

## Supplementary information

Supplementary Information
