# Supplementary material for: Carbonized polymer dots enhanced stability and flexibility of quasi-2D perovskite photodetector
Source: Light Sci Appl. 2022 Oct 18;11:304. doi: 10.1038/s41377-022-01000-6 (PMC9579173; doi:10.1038/s41377-022-01000-6)
Supplement: Supplementary file 1 — Supplementary Information [file 41377_2022_1000_MOESM1_ESM.docx]

**Supplementary Information for**

**Carbonized Polymer Dots Enhanced Stability and Flexibility of Quasi-2D Perovskite Photodetector**

*Mingrui Tan^1^, Mingbian Li^1^, Wanting Pan^1^, Xiaopeng Feng^1^, Yuhong He^1^, Junjun Liu^2^, Fengxia Dong^1^, Haotong Wei^1,2^[[1]](#footnote-1)^*^, and Bai Yang^1,2^*

^1^State Key Laboratory of Supramolecular Structure and Materials, College of Chemistry, Jilin University, Changchun, 130012, China

^2^ Optical Functional Theranostics Joint Laboratory of Medicine and Chemistry, The First Hospital of Jilin University, Changchun, 130012, China

**Supplementary Figures**


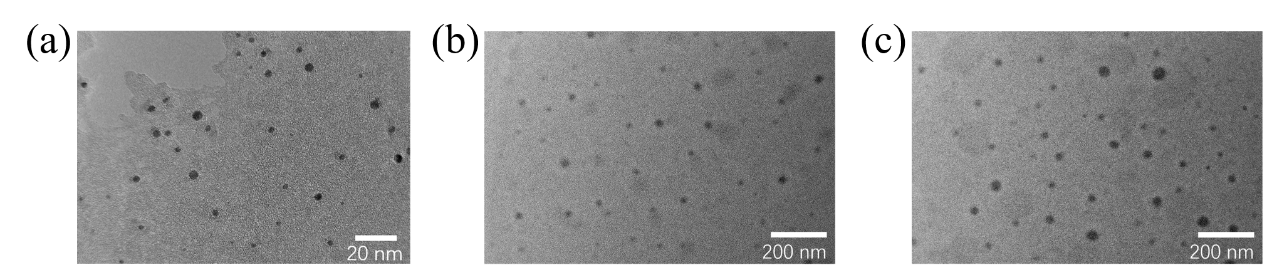


**Figure S1**. The TEM images of CPDs cores fabricated at 120℃ (a), 150℃ (b), and 180℃ (c).

Figure S1 shows the Transmission Electron Microscope (TEM) images of three prepared CPDs with carbonazation temperatures of 120℃, 150℃ and 180℃. The statistic was analysed in Figure 1b in the discussion part.


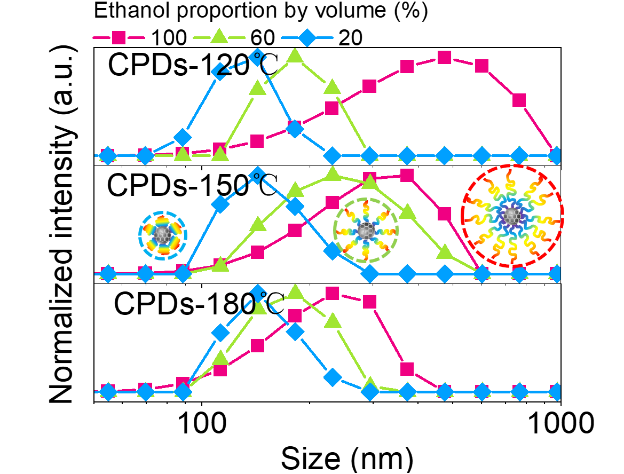


**Figure S2**. The DLS measurement results of various CPDs. The solvent was prepared by mixing ethanol and deionized water under different volume ratios.

The morphology information of three CPDs was further quantified via Dynamic Light Scattering (DLS) measurements, as seen in Figure S2. We vary the solvent mixture comprising ethanol as a good solvent and deionized water as a poor solvent, to manipulate the distribution properties of CPDs' polymer chain. As the inset indicates, the polymer chains stretch out in pure ethanol as schematic in the red circle. The CPDs-120℃ have the longest polymer chain within about 500 nm. With gradually increasing the proportion of poor solvent, the polymer chains continuously shrink and finally contract close to the carbon core, as schematic in the blue and green circles, respectively. The final polymer chain-core added size was 130 nm, 140 nm, and 145 nm, for CPDs-120℃, CPDs-150℃, and CPDs-180℃, respectively.


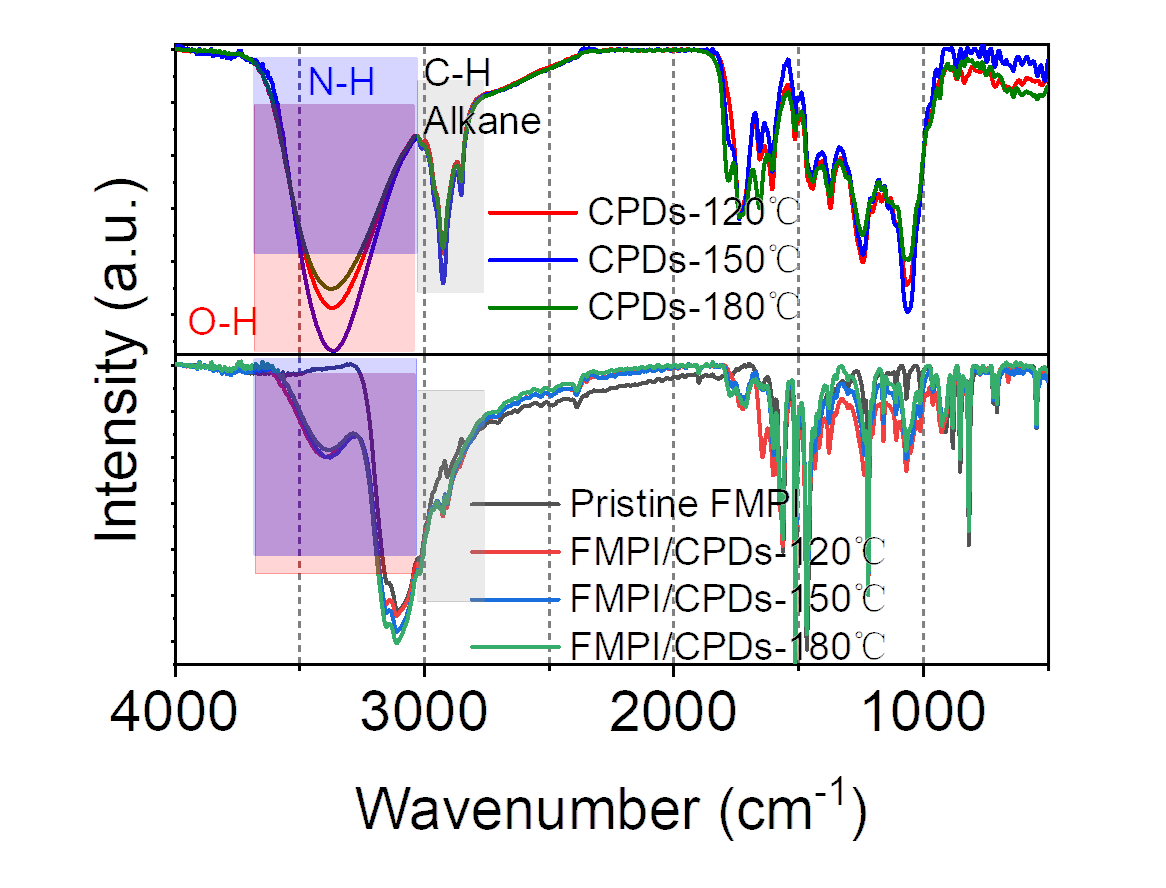


**Figure S3**. The FTIR results for three different prepared CPDs (top) and FMPI/CPDs samples (bottom).

The functional groups' species contained in different CPDs were identified via Fourier Transform Infrared Spectroscopy (FTIR). As seen in Figure S3, all the CPDs contain aminos, hydroxyls, and alkanes groups at the single bond stretching vibration region. At the double bond region, the characteristic peaks of amides, aromatics, carboxylic acids, anhydrides appear as components of the CPDs, and carboxylic acids, amides, and ether bonds show up in the fingerprint region, as seen in Figure 1d in the discussion part.


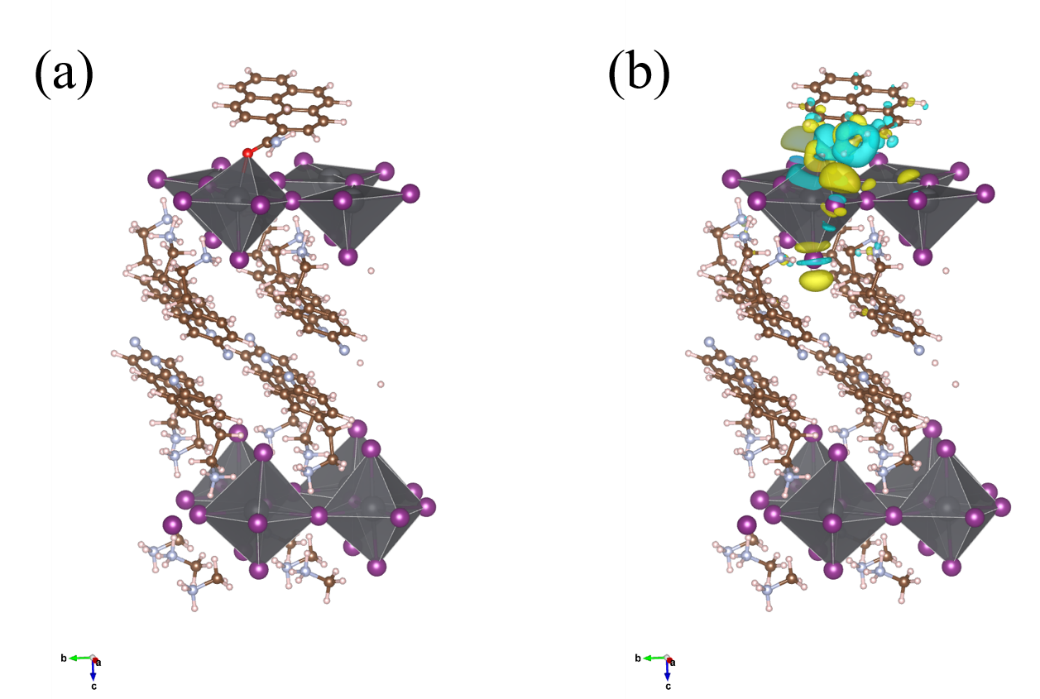


**Figure S4**. The optimized structure (a) and the DC analyses (b) of CPDs with amides group coordinate with unbonded Pb atom.


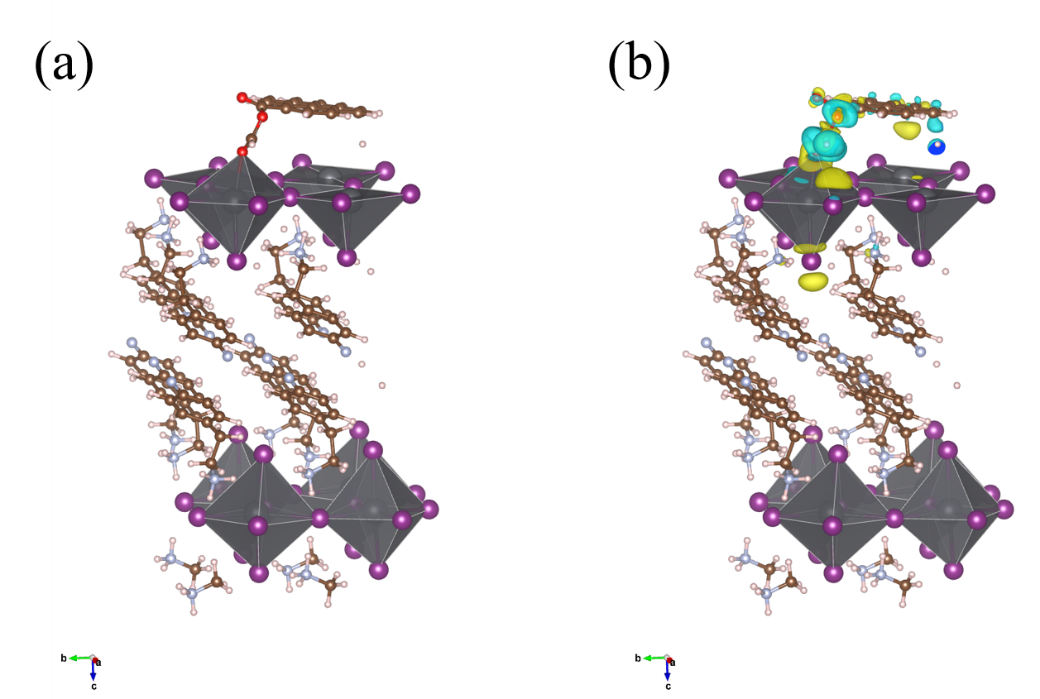


**Figure S5**. The optimized structure (a) and the DC analyses (b) of CPDs with anhydrides group coordinate with unbonded Pb atom.

Figure S4a and S5a show the optimized structure of the amides group and anhydrides group ended CPDs coordinated with the unbonded Pb atoms separately. Figure S4b and S5b shows the differential charge analyses results of both optimized structures, respectively. The yellow area represents the electrons accumulating process, while the blue area is losing. Obviously, both carbonyl-contained groups have a charge transfer phenomenon that the electron transfer from the coordinated Pb atom to the O atom belonging to the carbonyl group. The simulating results are similar to carboxylic acids ended CPDs coordinated structure.


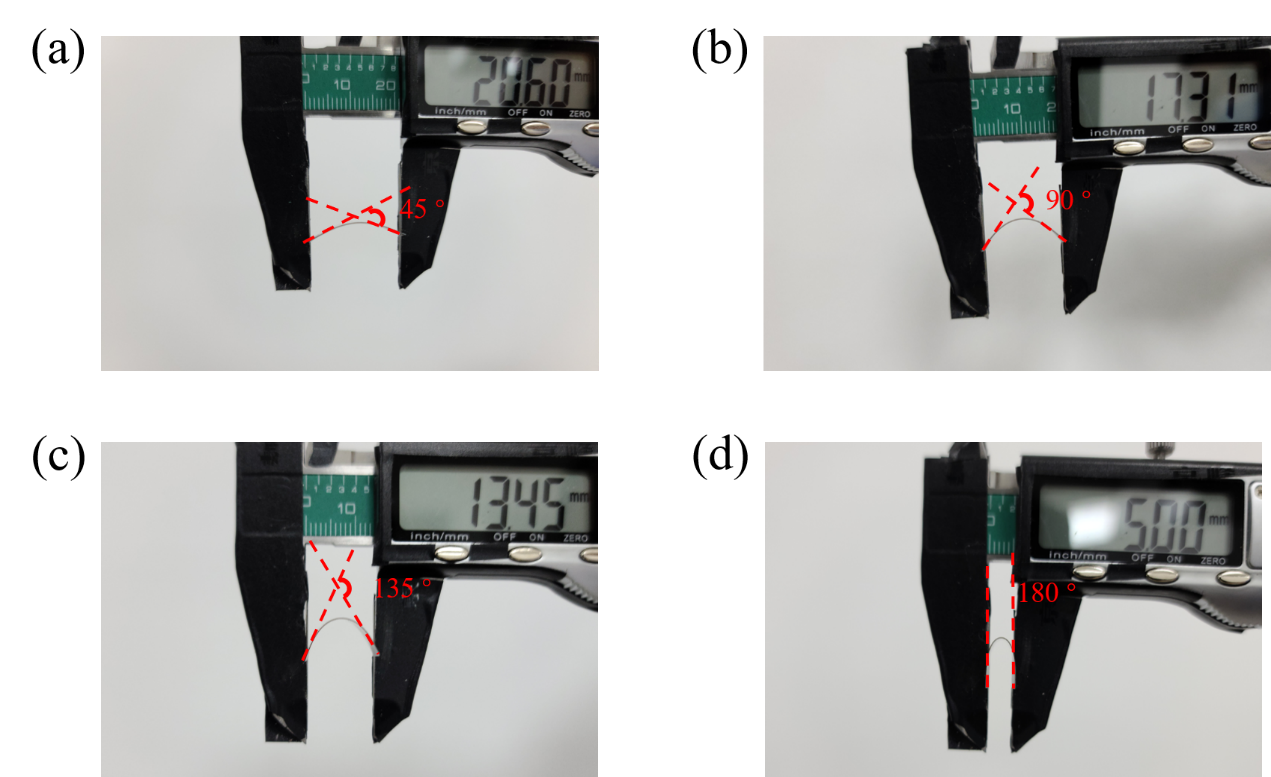


**Figure S6**. The scheme of bending angle stability measurements versus different bending degrees for 45° (a), 90° (b), 135° (c), and 180° (d).

To clearly quantify the bending grades, we give a group of photos to show our experiment condition setting. Figure S6a, b, c, and d represents a bending degree of 45°, 90°, 135°, and 180°, respectively. The actual bending degree was controlled by our homemade motor device, as described in the discussion part.


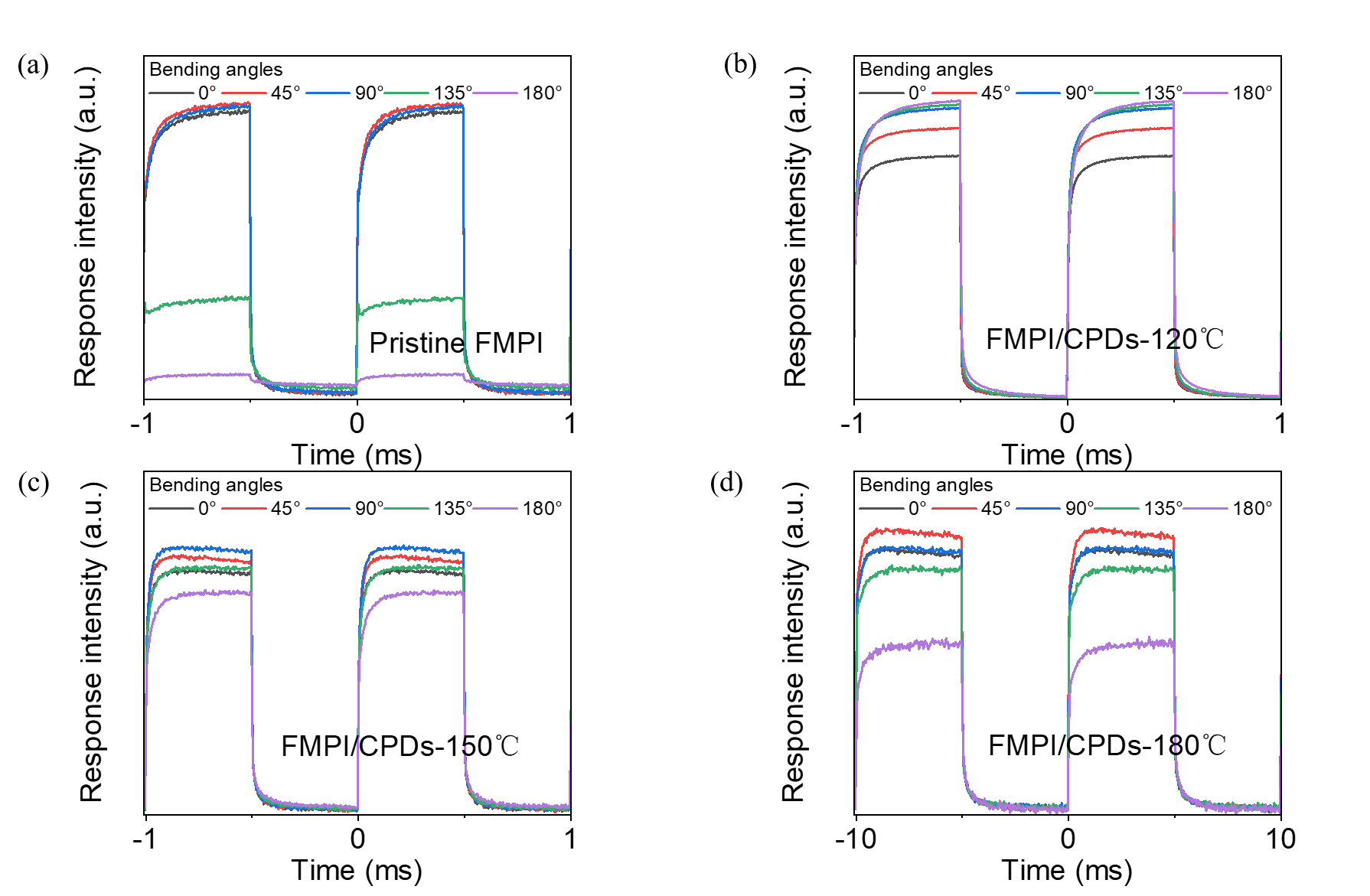


**Figure S7.** The response of on-off measurements of pristine FMPI (a), FMPI/CPDs-120℃ (b), FMPI/CPDs-150℃ (c), and FMPI/CPDs-180℃ (d) versus various bending angles.

Figure S7 shows the original data of the flexible stability tests, measured after bending 1000 times under various angles. As seen in Figure S7a, the response signal of the pristine FMPI device shows an obvious reduction after bending 135°, while all three FMPI/CPDs devices show better response maintenance. Most importantly, with the polymer chains lengthened, the response signal has a better retaining that the FMPI/CPDs-120℃ device shows no loss even after bending 1000 times with an angle of 180°. The statistical results were given in Figure 3b in the discussion part.


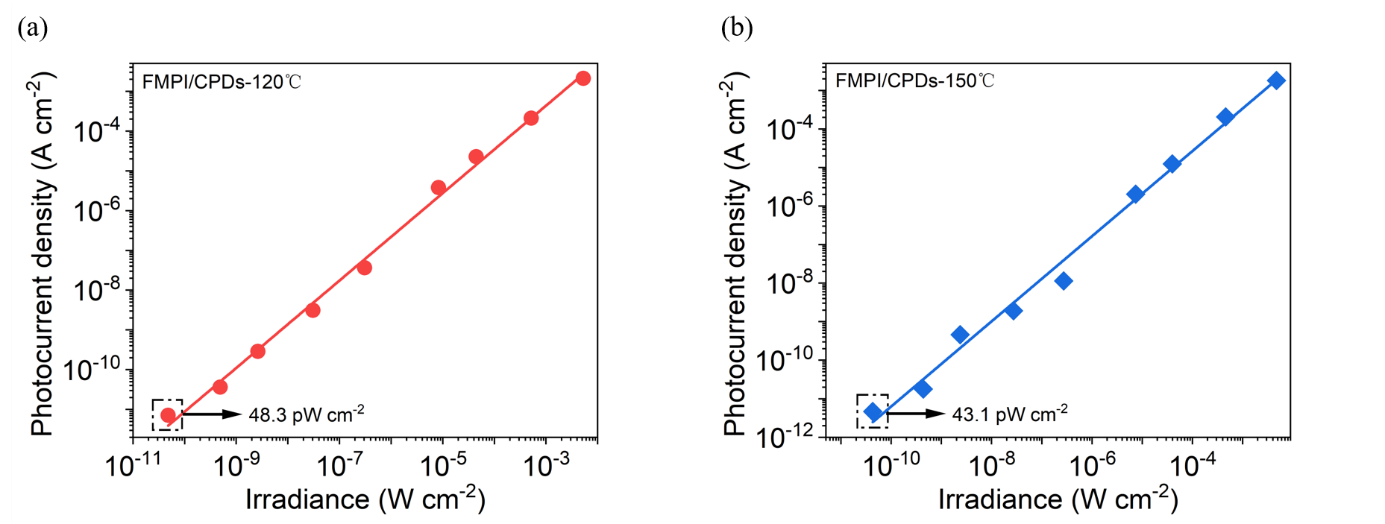


**Figure S8.** The NEP measurement results of FMPI/CPDs-120℃ and FMPI/CPDs-150℃.

As shown in Figure S8, the detection limit of FMPI/CPDs-120℃ and FMPI/CPDs-150℃ is 48.3 and 43.1 pw cm^-2^ obtained via NEP measurements. As the detection limits of pristine FMPI and FMPI/CPDs-180℃ are 542.4 and 10.1 pw cm^-2^ in the manuscript, separately, we can conclude that all the CPDs promote the detection performance for weak intensity light, as they all decrease the dark current of the photodetectors. The CPDs 180℃ has the lowest detection limit, keeping consistent with the manuscript.

**Table S1.** Flexible devices bending stability.

| Material | Bending cycles (times) | Response retaining (%) |  | Reference |
| --- | --- | --- | --- | --- |
| MoS_2_ | 500 | 100 | ^1^ | |
|  | 500 | 100 | ^2^ | |
|  | 1000 | 80 | ^3^ | |
|  | 10000 | 71 | ^4^ | |
|  | 10000 | 94 | ^5^ | |
| Graphene | 1000 | 80 | ^6^ | |
|  | 3500 | 97 | ^7^ | |
|  | 5000 | 100 | ^8^ | |
|  | 25000 | 10 | ^9^ | |
|  | 100 | 45 | ^10^ | |
|  | 1000 | 100 | ^11^ | |
| Metal oxide | 200 | 100 | ^12^ | |
|  | 200 | 100 | ^13^ | |
|  | 1000 | 100 | ^14^ | |
|  | 2000 | 100 | ^15^ | |
| Perovskite | 12000 | 80 | ^16^ | |
|  | 1000 | 75 | ^17^ | |
|  | 1000 | 76 | ^18^ | |
|  | 1000 | 105 | ^19^ | |
|  | 9000 | 105 | ^20^ | |
|  | 9000 | 82 | ^21^ | |
|  | 10000 | 90 | ^22^ | |
|  | 10000 | 1 | ^23^ | |
|  | 5000 | 80 | ^24^ | |
|  | 1000 | 90 | ^25^ | |
|  | 1000 | 100 | ^26^ | |
|  | 5000 | 95 | ^27^ | |
|  | 1000 | 98 | ^28^ | |
|  | 1000 | 100 | ^29^ | |
| Polymer | 6000 | 75 | ^30^ | |
|  | 1000 | 98 | ^31^ | |
|  | 1000 | 90 | ^32^ | |
|  | 100 | 90 | ^33^ | |
|  | 1500 | 96 | ^34^ | |
|  | 150 | 85 | ^35^ | |
|  | 1000 | 84 | ^36^ | |
|  | 91 | 102 | ^37^ | |
| Composite | 3000 | 30 | ^38^ | |
|  | 10000 | 94 | ^39^ | |
|  | 2000 | 87 | ^40^ | |
|  | 10000 | 100 | ^41^ | |
|  | 20000 | 91 | ^42^ | |
|  | 10000 | 85 | ^43^ | |
|  | 10000 | 74 | ^44^ | |
|  | 10000 | 76 | ^44^ | |
|  | 3000 | 100 | ^45^ | |
|  | 7000 | 92 | ^46^ | |
|  | 10000 | 100 | ^23^ | |
|  | 1000 | 92 | ^47^ | |
|  | 1000 | 82 | ^48^ | |
|  | 5000 | 85 | ^49^ | |
|  | 2000 | 84 | ^50^ | |
|  | 5000 | 85 | ^51^ | |
|  | 10000 | 97 | ^52^ | |
|  | 1000 | 98 | ^53^ | |
|  | 100 | 88 | ^54^ | |
| Perovskite/CPDs | 10000 | 110% | This work | |

Table S1 lists the reported flexible optoelectronic devices with their retaining response after the bending process. The summary was illustrated in Figure 3d.

**References**

1. Selamneni, V., Raghavan, H., Hazra, A. & Sahatiya, P. MoS_2_ Paper Decorated with Metal Nanoparticles (Au, Pt, and Pd) Based Plasmonic‐Enhanced Broadband (Visible‐NIR) Flexible Photodetectors. *Advanced Materials Interfaces* **8**, 2001988 (2021).

2. Gomathi, P.T., Sahatiya, P. & Badhulika, S. Large-Area, Flexible Broadband Photodetector Based on ZnS-MoS2 Hybrid on Paper Substrate. *Advanced Functional Materials* **27**, 1701611 (2017).

3. Pak, S. et al. Surface functionalization-induced photoresponse characteristics of monolayer MoS2 for fast flexible photodetectors. *Nanoscale* **11**, 4726-4734 (2019).

4. Sun, B. et al. Tailoring of Silver Nanocubes with Optimized Localized Surface Plasmon in a Gap Mode for a Flexible MoS_2_ Photodetector. *Advanced Functional Materials* **29**, 1900541 (2019).

5. Lim, Y.R. et al. Wafer-Scale, Homogeneous MoS2Layers on Plastic Substrates for Flexible Visible-Light Photodetectors. *Advanced Materials* **28**, 5025-5030 (2016).

6. Zhang, Y. et al. Graphdiyne‐Based Flexible Photodetectors with High Responsivity and Detectivity. *Advanced Materials* **32**, 2001082 (2020).

7. Liu, Y.-L. et al. Transparent, Broadband, Flexible, and Bifacial-Operable Photodetectors Containing a Large-Area Graphene–Gold Oxide Heterojunction. *ACS Nano* **9**, 5093-5103 (2015).

8. Sun, T. et al. Flexible Broadband Graphene Photodetectors Enhanced by Plasmonic Cu_3−x_P Colloidal Nanocrystals. *Small* **13**, 1701881 (2017).

9. Wang, Q.-M. & Yang, Z.-Y. Graphene photodetector with polydiacetylenes acting as both transfer-supporting and light-absorbing layers: Flexible, broadband, ultrahigh photoresponsivity and detectivity. *Carbon* **138**, 90-97 (2018).

10. Chong, W.G. et al. Lithium-Sulfur Battery Cable Made from Ultralight, Flexible Graphene/Carbon Nanotube/Sulfur Composite Fibers. *Advanced Functional Materials* **27**, 1604815 (2017).

11. Das, S. et al. A leaf-inspired photon management scheme using optically tuned bilayer nanoparticles for ultra-thin and highly efficient photovoltaic devices. *Nano Energy* **58**, 47-56 (2019).

12. Zheng, Z., Gan, L., Zhang, J., Zhuge, F. & Zhai, T. An Enhanced UV-Vis-NIR an d Flexible Photodetector Based on Electrospun ZnO Nanowire Array/PbS Quantum Dots Film Heterostructure. *Advanced Science* **4**, 1600316 (2017).

13. Zheng, Z. et al. A Fully Transparent and Flexible Ultraviolet-Visible Photodetector Based on Controlled Electrospun ZnO-CdO Heterojunction Nanofiber Arrays. *Advanced Functional Materials* **25**, 5885-5894 (2015).

14. Wang, B. et al. Flexible and stretchable metal oxide nanofiber networks for multimodal and monolithically integrated wearable electronics. *Nature Communications* **11** (2020).

15. Li, L., Gu, L., Lou, Z., Fan, Z. & Shen, G. ZnO Quantum Dot Decorated Zn2SnO4 Nanowire Heterojunction Photodetectors with Drastic Performance Enhancement and Flexible Ultraviolet Image Sensors. *ACS Nano* **11**, 4067-4076 (2017).

16. Asuo, I.M. et al. Highly Efficient and Ultrasensitive Large-Area Flexible Photodetector Based on Perovskite Nanowires. *Small* **15**, 1804150 (2019).

17. Wang, M., Sun, H., Cao, F., Tian, W. & Li, L. Moisture‐Triggered Self‐Healing Flexible Perovskite Photodetectors with Excellent Mechanical Stability. *Advanced Materials*, 2100625 (2021).

18. Tong, G. et al. Dual-Phase CsPbBr_3_-CsPb_2_Br_5_ Perovskite Thin Films via Vapor Deposition for High-Performance Rigid and Flexible Photodetectors. *Small* **14**, 1702523 (2018).

19. Leung, S.-F. et al. A Self-Powered and Flexible Organometallic Halide Perovskite Photodetector with Very High Detectivity. *Advanced Materials* **30**, 1704611 (2018).

20. Lai, Z. et al. High‐Performance Flexible Self‐Powered Photodetectors Utilizing Spontaneous Electron and Hole Separation in Quasi‐2D Halide Perovskites. *Small* **17**, 2100442 (2021).

21. Dong, R., Lan, C., Li, F., Yip, S. & Ho, J.C. Incorporating mixed cations in quasi-2D perovskites for high-performance and flexible photodetectors. *Nanoscale Horizons* **4**, 1342-1352 (2019).

22. Deng, H. et al. Flexible and Semitransparent Organolead Triiodide Perovskite Network Photodetector Arrays with High Stability. *Nano Lett* **15**, 7963-7969 (2015).

23. Yoon, J. et al. Foldable Perovskite Solar Cells Using Carbon Nanotube‐Embedded Ultrathin Polyimide Conductor. *Advanced Science* (2021).

24. Hu, X. et al. Nacre-inspired crystallization and elastic “brick-and-mortar” structure for a wearable perovskite solar module. *Energy & Environmental Science* **12**, 979-987 (2019).

25. Park, M. et al. Mechanically Recoverable and Highly Efficient Perovskite Solar Cells: Investigation of Intrinsic Flexibility of Organic-Inorganic Perovskite. *Advanced Energy Materials* **5**, 1501406 (2015).

26. Kang, S. et al. Ultrathin, lightweight and flexible perovskite solar cells with an excellent power-per-weight performance. *Journal of Materials Chemistry A* **7**, 1107-1114 (2019).

27. Li, Y. et al. High-efficiency robust perovskite solar cells on ultrathin flexible substrates. *Nature Communications* **7**, 10214 (2016).

28. Li, H. et al. Ultraflexible and biodegradable perovskite solar cells utilizing ultrathin cellophane paper substrates and TiO2/Ag/TiO2 transparent electrodes. *Solar Energy* **188**, 158-163 (2019).

29. Chung, J. et al. Record-efficiency flexible perovskite solar cell and module enabled by a porous-planar structure as an electron transport layer. *Energy & Environmental Science* **13**, 4854-4861 (2020).

30. Chen, X. et al. Realizing Ultrahigh Mechanical Flexibility and >15% Efficiency of Flexible Organic Solar Cells via a “Welding” Flexible Transparent Electrode. *Advanced Materials* **32**, 1908478 (2020).

31. Yan, T. et al. 16.67% Rigid and 14.06% Flexible Organic Solar Cells Enabled by Ternary Heterojunction Strategy. *Advanced Materials* **31**, 1902210 (2019).

32. Zhang, Z. et al. Integrated Polymer Solar Cell and Electrochemical Supercapacitor in a Flexible and Stable Fiber Format. *Advanced Materials* **26**, 466-470 (2014).

33. Chen, S. et al. Highly Flexible and Efficient All‐Polymer Solar Cells with High‐Viscosity Processing Polymer Additive toward Potential of Stretchable Devices. *Angewandte Chemie International Edition* **57**, 13277-13282 (2018).

34. Zhang, J. et al. Self-Doping Fullerene Electrolyte-Based Electron Transport Layer for All-Room-Temperature-Processed High-Performance Flexible Polymer Solar Cells. *Advanced Functional Materials* **28**, 1705847 (2018).

35. Kim, T. et al. Flexible, highly efficient all-polymer solar cells. *Nature Communications* **6**, 8547 (2015).

36. Kim, Y.U. et al. Optimal Design of PEDOT:PSS Polymer-Based Silver Nanowire Electrodes for Realization of Flexible Polymer Solar Cells. *Macromolecular Research* **29**, 75-81 (2021).

37. Salinas, J.-F. et al. Optical Design of Transparent Thin Metal Electrodes to Enhance In-Coupling and Trapping of Light in Flexible Polymer Solar Cells. *Advanced Materials* **24**, 6362-6367 (2012).

38. Zhou, G., Sun, R., Xiao, Y., Abbas, G. & Peng, Z. A High‐Performance Flexible Broadband Photodetector Based on Graphene–PTAA–Perovskite Heterojunctions. *Advanced Electronic Materials* **7**, 2000522 (2021).

39. Li, X. et al. Constructing Fast Carrier Tracks into Flexible Perovskite Photodetectors To Greatly Improve Responsivity. *ACS Nano* **11**, 2015-2023 (2017).

40. Ko, J.S. et al. All-two-dimensional semitransparent and flexible photodetectors employing graphene/MoS2/graphene vertical heterostructures. *Journal of Alloys and Compounds* **864**, 158118 (2021).

41. Dang, V.Q. et al. High-Performance Flexible Ultraviolet (UV) Phototransistor Using Hybrid Channel of Vertical ZnO Nanorods and Graphene. *ACS Applied Materials & Interfaces* **7**, 11032-11040 (2015).

42. Sun, B. et al. Sensitive, fast, and stable photodetector based on perovskite/MoS2 hybrid film. *Applied Surface Science* **493**, 389-395 (2019).

43. Kang, M.-A. et al. Fabrication of flexible optoelectronic devices based on MoS2/graphene hybrid patterns by a soft lithographic patterning method. *Carbon* **116**, 167-173 (2017).

44. Lee, Y.B. et al. Dimensional-Hybrid Structures of 2D Materials with ZnO Nanostructures via pH-Mediated Hydrothermal Growth for Flexible UV Photodetectors. *ACS Applied Materials & Interfaces* **9**, 15031-15037 (2017).

45. Dang, V.Q. et al. Methylammonium lead iodide perovskite-graphene hybrid channels in flexible broadband phototransistors. *Carbon* **105**, 353-361 (2016).

46. Yue, Y. et al. A Flexible Integrated System Containing a Microsupercapacitor, a Photodetector, and a Wireless Charging Coil. *ACS Nano* **10**, 11249-11257 (2016).

47. Xu, X. et al. Surface Functionalization of a Graphene Cathode to Facilitate ALD Growth of an Electron Transport Layer and Realize High-Performance Flexible Perovskite Solar Cells. *ACS Applied Energy Materials* **3**, 4208-4216 (2020).

48. Heo, J.H., Shin, D.H., Lee, M.L., Kang, M.G. & Im, S.H. Efficient Organic–Inorganic Hybrid Flexible Perovskite Solar Cells Prepared by Lamination of Polytriarylamine/CH_3_NH_3_PbI_3_/Anodized Ti Metal Substrate and Graphene/PDMS Transparent Electrode Substrate. *ACS Applied Materials & Interfaces* **10**, 31413-31421 (2018).

49. Heo, J.H. et al. Super-flexible bis(trifluoromethanesulfonyl)-amide doped graphene transparent conductive electrodes for photo-stable perovskite solar cells. *Journal of Materials Chemistry A* **6**, 8251-8258 (2018).

50. Luo, Q. et al. All-Carbon-Electrode-Based Endurable Flexible Perovskite Solar Cells. *Advanced Functional Materials* **28**, 1706777 (2018).

51. Yoon, J. et al. Superflexible, high-efficiency perovskite solar cells utilizing graphene electrodes: towards future foldable power sources. *Energy & Environmental Science* **10**, 337-345 (2017).

52. Jeong, G. et al. Suppressed Interdiffusion and Degradation in Flexible and Transparent Metal Electrode-Based Perovskite Solar Cells with a Graphene Interlayer. *Nano Letters* **20**, 3718-3727 (2020).

53. Wu, S. et al. Low‐Bandgap Organic Bulk‐Heterojunction Enabled Efficient and Flexible Perovskite Solar Cells. *Advanced Materials*, 2105539 (2021).

54. La Notte, L. et al. Fully-sprayed flexible polymer solar cells with a cellulose-graphene electrode. *Materials Today Energy* **7**, 105-112 (2018).

1. *Correspondence to H.W. at Email: [hweichem@jlu.edu.cn](mailto:hweichem@jlu.edu.cn) [↑](#footnote-ref-1)
